# Factors affecting early re-displacement of paediatric diaphyseal forearm fractures at Korle Bu Teaching Hospital

**DOI:** 10.4314/gmj.v54i3.5

**Published:** 2020-09

**Authors:** Daniel Y Agbley, Henry A Holdbrook-Smith, Michael Segbefia, Yao Ahonon, Kissinger Marfo

**Affiliations:** 1 University of Health and Allied Sciences, Department of Surgery, Ho Teaching Hospital, Ho, Ghana; 2 Department of Trauma and Orthopaedic, Korle Bu Teaching Hospital, Korle-Bu-Accra, Ghana; 3 Public Health Unit, Korle Bu Teaching Hospital, Korle Bu, Accra, Ghana

**Keywords:** cast index, intermedullary nailing, elastic stable intramedullary nail, open reduction, internal fixation

## Abstract

**Background:**

Complete fractures of the forearm have the potential to displace and angulate with overriding fracture fragments. Maintaining acceptable reduction is not always possible, and re- displacement or re-angulation is the most commonly reported complication. Factors responsible for the re-displacement after an initial acceptable reduction have not been clearly defined. The study aimed to determine the factors that influence early re-displacement of paediatric diaphyseal forearm fractures in Korle-Bu Teaching Hospital.

**Methods:**

A prospective study in a cohort of 72 children below the age of 12 years with diaphyseal forearm fracture attending the Orthopaedic clinic were followed with close reduction casting from April 2017-December, 2017. Factors analysed included demographics, initial fracture features and the radiographic indices of the cast quality.

**Results:**

93.1% (67) of the fractures were because of the children falling on an outstretched arm. Majority of the children had a fracture of the distal 1/3 of the radius (n=38, 52.6%). The overall C.I was 0.8 (SD 0.1). The only significant predictor for predicting re-displacement was children falling on an outstretched hand (p-value=0.0).

**Conclusion:**

This study has shown that the degree of initial displacement and the ability to achieve good reduction with a well moulded cast, constitute the major factors for early re-displacement of paediatric forearm fractures

**Funding:**

Personal funding

## Introduction

Diaphyseal or shaft fractures of the forearm are fractures occurring between the proximal and distal metaphysis of the either or both ulna and radius. The metaphysis is the diverging area of the bone between the diaphysis (shaft) and the physis (growth plate) radius and ulna naturally heal rapidly with an excellent functional outcome if the reduction is maintained. Forearm fractures are one of the most common fractures occurring in children. It accounts for 39% of all pediatric fractures^1^, which commonly arises as a result of a fall on an outstretched arm. Today, forearm fractures are often managed conservatively with closed reduction and cast immobilisation. Successful treatment of fractures results in restoration of anatomic alignment and full recovery of range of motion.^2^ However, complications do occur, and one of the most frequently occurring complications is early redisplacement of the reduced fracture. Early redisplacement is a displacement of a fracture within two weeks after closed reduction and cast immobilisation which leads to malunion. This malunion subsequently results in a restricted range of motion of the forearm.^3^ Studies have shown that early redisplacement occurs in 12% to 34% of cases after the treatment.^4^ Recently, there has been an increase in the incidence of redisplacement fracture after treatment^5^,^6^, especially those with early instability. In order to reduce the incidence of redisplacement, information regarding risk factors are essential. This because fracture risk stratification allows early detection of highly unstable fractures that may be susceptible to displacement fracture and may require other forms of intervention such as K-wire fixation or Open reduction and internal fixation (ORIF). Many studies have evaluated factors associated with displacement fracture after reduction and immobilisation treatment.^2^,^4^,^7^

These risk factors have been classified into patient-related, fracture-related and treatment or surgeon-related factors.^3^

For example, the severity of the initial displacement, a fracture -related factor, has been linked to redisplacement.^4^ However, little prospective data exist as regards risk factors associated with fracture redisplacement and level of diaphyseal forearm fracture. The best research design to identify potential risk factors is a prospective study that involves a cohort of children. To date, there is no consensus about which of the risk factors are clinically relevant. We conducted a prospective study in a cohort of children attending the Orthopaedic clinic at Korle-Bu Teaching Hospital to determine the factors that are associated with early redisplacement and level of diaphyseal forearm fracture.

## Methods

### Setting and Patient selection

This was a prospective study design and was undertaken in the Department of Trauma and Orthopaedics of the Korle Bu Teaching Hospital which attends to about 22,355 out-patient's cases yearly. Seventy children who are below 12 years with close displaced and undisplaced radius and ulna, radius or ulna fracture were recruited for the study from April 2017 to December 2017. Patients were excluded if they had open forearm fractures, green-stick fractures, plastic deformed fractures, segmental fractures, Galeazzi fractures, monteggia fractures, floating elbow, pathological fractures, multiple injured patients. Additional exclusions were patients with previous fracture of the same forearm and patients presenting after 1 week of injury.

### Procedure

A structured evaluation data collection sheet was administered to all the consenting patients whose children met the inclusion criteria. Patients were managed non-operatively with above elbow cast, and the cast index measured. The cast index is a ratio of anteroposterior to lateral internal diameters of the cast at the fracture site. Cast index of more than 0.7 correlates with increased risk of redisplacement. All fractures were manipulated to the anatomical position under sedation and analgesia before application of above elbow cast with elbow flexed at 90 degrees and forearm kept in the neutral position in distal third radius and ulna fractures, pronation in middle third fractures and in supination for proximal third fractures without the aid of image intensifier. Three-point fixation was applied (Fracture is reduced, plaster cast is moulded to apply pressure at the site of the fracture and proximal/distal to the fracture). Post reduction check X-ray is done to confirm the anatomical reduction before patient is discharged. Patients were followed up in the clinic one week after manipulation and assess for re-displacement with x-ray and cast index measurement, and then followed up every week until fracture union and cast removed in six weeks.

Functional outcome (pronation and supination, angular and rotational deformity and translation) was assessed using the grading system for functional outcome by Price et al in three months and six months' period of follow up. Re-displacement was considered if there is more than 10 degrees dorsal or volar angulation, more than 5 degrees of radial deviation, more than 3mm of translation or combination of more than 2mm of translation and more than 5 degrees of angulation.

Fracture re-manipulation was done in cases of more than 20 degrees dorsal angulation alone, more than 10 degrees radial deviation alone or 4mm of translation alone and combination of at least two of the following (more than 10 degrees of dorsal angulation, more than 5 degrees of radial deviation and more than 3mm of translation). Patients in whom maintaining the initial reduction fails and those that the fracture re-displaces after second re-manipulation exited the study.

### Data Analysis

Statistical Analysis was performed using SPSS Quantitative variables are presented as mean (SD). Qualitative variables as percentages. Possible predictors that correlated with re-displacement were analysed using forward logistic regression techniques. Odds ratio (OR) associated with each factor measured and 95% confidence interval (CI) were calculated. All p-values were based on 2-tailed tests and a probability value of ≤ 0.05 was considered statistically significant.

### Ethics

The study was reviewed and approved by The Research and Ethical Review Committee of the College of Health Sciences and written informed consent was obtained from the patients or wards before the investigation (date of issue: April 04, 2017; protocol identification number: CHS-Et/M.7 – P3.3/2016-2017).

## Results

A total of 72 children were fulfilled the inclusion criteria and were entered into the study. More than half of the children were below the age of 9 years (83.3%). The average age at injury was 6.2 years (SD 3). There were 53 males (73.6%) and 19 females (26%) as shown in Table 1. At presentation, the right hand was the dominant hand of the children (58, 80.6%). Most of the injury were as a result of the children falling on an outstretched arm (67, 93.1%). 41 children had right side fracture (56.9%) whereas the rest had left side fracture (31, 43.1%).

**Table 1:** Demographic profile of the children involved in the study

Variable	Frequency n (%)
**Age group**	
**<9 years**	60 (83.3)
**> 9 years**	12 (16.7)
**Mean age ± SD**	
**Gender**	
**Male**	53 (74)
**Female**	19 (26)
**Dominant hand**	
**Right hand**	58 (81)
**Left hand**	14 (19)
**Mechanism of Injury**	
**Fallen on outstretched arm**	67 (93)
**Sport injury**	3 (4)
**Direct blow**	1 (1)
**Road Traffic Accident**	1 (1)
**Side of Fracture**	
**Left**	31 (43)
**Right**	41 (57)
**Level of Fracture**	
**Distal 1/3**	38 (53)
**Middle 1/3**	27 (38)
**Proximal 1/3**	7 (9)

A little above half of the children had distal 1/3 level of fracture (38, 52.6%), 27 (37.5%) had middle 1/3 level and 7 (9.9%) had proximal 1/3 level as shown in Table 1. The average cast index was 0.81 (SD 0.1)

Almost all of the children presented with a transverse pattern of fracture (70, 97.2%) and the rest was Oblique pattern of fracture. Forty-eight (66.7%) children had incomplete fracture, whereas the rest of the children had a complete fracture. 53 (73.6%) children had volar direction of angulation, 16 (22.2%) dorsal and 3 were Radial (4.2%). Majority of the children came with a < 10 degree of angulation (68, 94.4%) and the rest were between 10 -15 degree of angulation.

Almost all of the children had shortening of <10 mm (70,97.2%), and the rest had a shortening > 10 mm. Seventy children had a translation of <50% whereas the rest had a translation between 50–100% as shown in Table 2. Fallen on an outstretched hand was the only predictive factor for displacement (p-value = 0.015). Furthermore, sex (p-value= 1.00), age group (p-value = 0.179), level of fracture (p-value = 0.603, 0.618, 1.000) and pattern of fracture (p-value = 0.539) are independent predictive factors for displacement as shown in Table 3.

**Table 2:** Radiographic Indices

Variable	Frequency n (%)
**Pattern of Fracture**	
**Transverse**	70 (97)
**Oblique**	2 (3)
**Direction of Angulation**	
**Volar**	53 (73.6)
**Dorsal**	16 (22.2)
**Radial**	3 (4.2)
**Nature of Fracture**	
**Complete**	24 (33)
**Incomplete**	48 (67)
**Degree of Angulation**	
**<10**	68 (94)
**10 – 15 Degrees**	4 (6)
**Shortening**	
**<10 mm**	70 (97)
**> 10 mm**	2 (3)
**Translation**	
**< 50%**	70 (97)
**50–100%**	2 (3)

**Table 3:** Regression of predictive factors for risk of displacement

Factors		Odds ratio	95% CI	p-value
**Sex**	Male	1.0	0.3–3.0	1.0
	Female	0.9	0.3–3.2	
**Age group**	< 9 years	2.5	0.7–9.0	0.2
	> 9 years	0.4	0.1–1.4	
**Mechanism** **of injury**	Fallen on an out- stretched hand	1.5	0.2–9.4	*0.0
	Sports injury	0.9	0.1–10.9	1.0
**level of fracture**	Distal 1/3	0.7	0.3–1.9	0.6
	Middle 1/3	1.3	0.5–3.6	0.6
	Proximal 1/3	1.2	0.2–6.7	1.0
**Pattern of** **fracture**	Transverse	2.2	0.1–36.5	0.5
	Oblique	0.5	0.0–7.7	

* statistically significant (p<0.05)

## Discussion

In this study, it has been shown that more males sustain forearm fracture as compare to female representing 73.6% males and 26.4% females. This result is comparable to the study done by Brudrik C et al that found males sustaining 59% of forearm fractures.^8^ The difference in male and female ratio is probably due to male children involving in contact sports and physical activities during break and closing times at school as compared to few females involving in physical activities.

Most of the paediatric forearm fractures in this study were found to occur in the dominant hand accounting for 56.9% and 31.43% in the non-dominant hand. This is consistent with the fact that most people falling from a height will fall with the dominant hand. This finding contradicts the study done by D Borton et al. which demonstrated that paediatric forearm fractures are common in the non-dominant hand.^9^ The mechanism of injury was commonly a fall on the outstretched arm (67.93.1%), this is true in most younger children who frequently jumped from a height as a window, a wall or a bed.

However, in older children, almost all fractures were due to sports activities or less frequently to other injuries. This study has shown that the most common sporting activity that led to forearm fracture in children below the ages of 9 years was jumping a see-saw trampoline which are found in most private schools in Ghana. Most of the fracture pattern in this study was transverse accounting for 70, 97.2%.

However, it did not affect the rate of re-displacement the forearm fractures.

In this study, the level of fracture was more common in the distal third of both radius and ulna (52.6%) followed by the middle third (37.5%) and least in the proximal third (9.9%). In accordance with other studies like a study done by Antabak et al^10^, this study proved that the distal third of the radius and ulna were the most common site of fracture. The direction of the deformity was predominantly volar in half of the cases (73.6%). The predominance of volar angulations is due to the mechanism of injury and the position of the hand at the time of the injury in which the arm was out stretched, the forearm pronated with the wrist dorsiflexed and the body rotated over the arm. The degree of angulation and the degree of rotation were all below 15 degrees.

Numerous authors have attempted to identify the risk factors involved in re-displacement after initial acceptable reduction and immobilisation in cast. There were many predictive factors used in this study, fallen on an outstretched hand which is a mechanism of injury was the only risk factor associated with displacement (OR=1.46, p-value=0.015).

This study was not able to establish patient age and gender as risk factors for fracture re-displacement. Similarly, Haddad et al^11^, Devaliya et al^12^ and Hang et al^13^ have reported that patient age and sex are not significant risk factors for re-displacement. This study had several limitations that should be considered. It is obvious that the sample size was small, especially patients above nine years, making it difficult to assess the risk of displacement in that study population.

Forearm fractures are common injuries in the paediatric population and successful treatment of these fractures should result in complication free functional pronation and supination. Cast index with its cut off point of less than 0.70, was not found to influence early re-displacement in this study, however, it can be used as a radiographic tool in evaluating a well moulded cast in the management of paediatric diaphyseal forearm fractures as other authors suggested.^14^ This study has shown that the degree of initial displacement and the ability to achieve good reduction with three-point fixation, constitute the major factors in preventing early re-displacement of paediatric forearm fractures.

## Conclusion

Forearm fractures are common injuries in the paediatric population and successful treatment of these fractures should result in complication-free functional pronation and supination. Non-operative management still remains the treatment of choice and a good cast application are a key to preventing early re-displacement.

This study had shown that the degree of initial displacement and the ability to achieve good reduction with threepoint fixation, constitute the major factors in preventing early re-displacement of paediatric forearm fractures.
